# Knowledge and Attitudes Towards Nutrigenetics: Findings from the 2019 Unified Forces Preventive Nutrition Conference (UFPN)

**DOI:** 10.3390/nu12020335

**Published:** 2020-01-27

**Authors:** Vered Kaufman-Shriqui, Hagit Salem, Mona Boaz, Ruth Birk

**Affiliations:** Department of Nutritional Sciences, School of Health Sciences, Ariel University, Ariel 40700, Israel; veredks@ariel.ac.il (V.K.-S.); hagitsa@ariel.ac.il (H.S.); monabo@ariel.ac.il (M.B.)

**Keywords:** dietitian, nutrigenomics, nutrigenetics, nutrition survey, education

## Abstract

Background: Nutrigenetics indicates that individual genetic variability results in altered health outcomes necessitating personalized nutrition adaptation. Registered dietitians are recognized as the clinical nutrition experts, but their knowledge and attitudes regarding nutrigenetics has not been delineated. Methods: This cross sectional online survey was conducted in a convenience sample of 169 national nutrition conference attendees. The survey queried demographics, knowledge, and attitudes towards nutrigenetics and information on training in nutrigenetics. Results: The majority of participants were registered dietitians and female, 45% of whom held advanced degrees. Personalized nutrition was perceived by 93.5% of participants as highly important or important; however, 94% of respondents indicated they are not sufficiently knowledgeable in personalized nutrition and only 9.5% had received training in nutrigenetics. The mean nutrigenetics knowledge score was 6.89 ± 1.67 (out of a possible 12). A multivariate regression model of knowledge score identified education as the only independent predictor of this outcome. Conclusion: Personalized nutrition is a rapidly developing field that incorporates genetic data into clinical practice. Dietitians recognize the importance of advanced studies to acquire knowledge in nutrigenetics. Only by acquiring the necessary knowledge can dietitians accurately translate this nutrigenetics into clinical practice.

## 1. Introduction

The post-genomic era is characterized by the rapid incorporation of genetic information into the fields of medicine and nutrition. The Human Genome Project, the 1000 Genomic Project, Genomics England and others have significantly advanced scientific knowledge regarding individual (personal) risk, pathophysiology, and treatment of common diseases [1,2,3]. In parallel, genetic information is increasingly introduced into the clinical setting, as well as to the scientific and clinical literature. Genetic tests are becoming an essential instrument in the toolbox of clinicians in medical and para-medical professions [4]. Additionally, genetic tests are marketed to the public through Direct-To-Customer companies, resulting in increasing consumer demand for interpretation and advice based on personal genetic information. Unfortunately, many clinical professionals, including registered dieticians, lack the knowledge and skills required to effectively deliver genetic services [5,6,7,8,9]. The challenges regarding the practical application of nutritional genomics were well demonstrated by a cross sectional online study by Collins et al., in which poor knowledge, involvement, and confidence in genetics and nutritional genomics was observed in 1844 dietitians from the US, Australia, and UK [10]. In light of this, the American Academy of Nutrition and Dietetics published a position paper [11] stating that: “registered dietitian nutritionists need basic competency in genetics as a foundation for understanding nutritional genomics; proficiency requires advanced knowledge and skills.” Due to the complexity of genetic information and the interaction with environmental factors, including nutrition, the position paper further specifies that: “applying nutritional genomics in clinical practice through the use of genetic testing requires that registered dietitian nutritionists understand, interpret, and communicate complex test results in which the actual risk of developing a disease may not be known.” Currently, although highly relevant to their profession, traditional dietitians’ curricula and/or training do not include extensive and wide training in advanced human genetics, including facets such as omics technology, interpretation of genetic variation information, as well as legal, ethical, and social aspects of genetic information, among others. Furthermore, in several countries, including Israel, dietitians are not legally entitled to counsel, preform, or treat patients using genetic information. 

Nutrigenetics or personalized nutrition is based on individual genetic variability that results in altered health outcome, and ultimately involves nutrition adjustment. As individual genetic information is stable from birth throughout life, and as its personal delineation has become feasible and relatively inexpensive, the incorporation of genetic information to routine medicine and nutrition/dietetics is becoming essential. For example, lactose intolerance, a common clinical phenotype worldwide (ranging from 0–90% of the population in different ethnicities), is caused by low to absent intestinal lactase activity, which results in a variety of symptoms, such as abdominal pain, flatulence, bloating and diarrhea, following lactose exposure. Consequently, many exclude milk and milk products (rich in lactose) from the diet, with consequent lack of calcium in the body and ultimately elevated risk of osteopenia and osteoporosis later in life [12,13]. Genetic single nucleotide polymorphisms (SNPs) (C>T-13910 and G>A-22018), located upstream to the lactase gene, have been strongly associated with adult lactose intolerance / persistence in several populations [14,15,16]. Due to the complexity and sensitivity of common biochemical and histological current methods of diagnosis, incorporating genetic screening for these common variations can significantly improve early diagnosis of lactase intolerance, enabling nutritional strategies to alleviate gastrointestinal symptoms in parallel to providing RDA calcium intake [17]. Despite these advancements, translation of nutrigenetics to the clinical setting is challenging and gradual and should be incorporated in light of restricted and evidence-based knowledge [18]. The potential impact of nutrigenetics has led to initiatives focused on changing consumer nutrition behavior, such as the EU-funded Food4Me project, which employs internet-delivered personalized nutrition (PN) [19,20,21,22]. 

Little is known regarding dietitians’ attitudes towards and knowledge of genetics and nutrigenomics. Existing data suggest that knowledge of nutrigenomics and nutrigenetics among dietitians is insufficient and varies across countries [10]. This study aims to assess the level of knowledge in genetics among health care professionals participating in a national Israeli nutrition conference and to estimate the associations between knowledge, sociodemographic characteristics, attitudes, and training in nutrigenomics.

## 2. Materials and Methods 

### 2.1. Overall Study Design and Plan

This cross sectional survey was conducted in a convenience sample of nutrition conference attendees. The survey queried demographics, knowledge, and attitudes towards nutrigenetics and information on professionals’ training in nutrigenetics. Investigators sent an online survey to all dietitians and other health professionals registered to participate in a large, national nutrition conference. 

### 2.2. Informed Consent

The present study was approved by the Institutional Ethics Committee (Helsinki Committee), Ariel University (AU-HEA-RB-20190523). Response to the survey indicated informed consent as stated on the first screen of the online study. 

### 2.3. Study Population 

All individuals who registered to attend the 17th Annual Preventive Nutrition—Combined Forces Israel Nutrition Week Conference received a link to an online survey administered via Google Surveys. Conference organizers indicated that approximately 3000 individuals registered for the event. As this conference is the largest annual nutrition conference in Israel, attended by dietitians, researchers, physicians, nurses, and other health professionals, the investigators selected this venue as a framework in which to query professionals in these fields. Conference organizers permitted researchers to utilize the registration lists as a target population. 

### 2.4. Inclusion Criteria

All individuals who were registered for the conference and responded to our email were sent a link to the online survey. The ability to respond to the survey, which was conducted in Hebrew, de facto included only individuals who are literate in Hebrew.

### 2.5. Facilitating Participation

To encourage participation in the survey, respondents were entered into a lottery for professionally meaningful prizes, including a digital scale and professional literature.

### 2.6. Survey Development

The survey included 24 questions surveying knowledge (12 multiple choice questions); training and attitudes in personalized nutrition (six questions, three on each topic); (File S1) and demographics (six questions). Demographic characteristics included personal information (age, sex, highest academic degree) and professional experience (profession, years in profession, employment setting). Demographic questions were mostly multiple choice (e.g., list of professions and employment settings), while report of years in profession and age were open questions. 

Knowledge questions on genetics and nutrigenetics were developed using a previously validated survey instrument, which was administered to dietitians in the US, UK, and Australia [10]. The questionnaire was translated to Hebrew, and then translated back to English by a different translator to ensure content validity. Most of the original questions were retained in the Hebrew questionnaire, and additional questions were added to reflect the current university-level introductory genetics course syllabus, which is compulsory for students studying towards an undergraduate degree in Nutrition Sciences in Israel. Knowledge questions were only related to nutrigenetics (Nutrigenetics or personalized nutrition is based on individual genetic (DNA) variability that results in individual nutrition recommendations).

A screening question examined participant training in PN counselling (incorporating genetic information into clinical nutrition practice). Respondents who replied positively were asked to indicate the duration of the course and, separately, the frequency of full-text research articles in nutrigenetics they read annually. These questions were also multiple choice. Finally, using a scale of 0–4, participants were asked to rate the importance of PN.

Once refined, face validity and online usability were determined through a pilot survey of 24 undergraduate nutrition students, and minor modifications were made based upon feedback. The survey was then graphically adapted for administration on mobile phones. Finally, five registered dietitians, providing expert validity, reviewed and approved the final version of the questionnaire. 

### 2.7. Data Analysis

SPSS v. 25.0 (IBM Inc., PASW Inc., Chicago, IL, USA) was used for all statistical analyses. Distributions of continuous variables were approximately normally distributed as assessed using the Kolmogorov-Smirnov test; thus, these are described using the mean ± standard deviation. Categorical variables such as the proportion of participants with a given response were described using frequency counts and expressed as *n* (%). Continuous variables were compared by profession using one-way analysis of variance (ANOVA). The associations between categorical variables were assessed using the chi- square test. The knowledge score was calculated as the sum of correct answers to the knowledge questions and ranged from 0–12. A multivariate regression model examined the association between sociodemographic characteristics of the participants and knowledge score (a score in the highest tertile vs. lower scores). All tests are two-sided and considered significant at *p* < 0.05. 

## 3. Results

Table 1 describes the survey participants. The majority of participants were registered dietitians and female. The areas of study for the highest degree of education included nutrition (53.8%); public health (11.2%); management (7.7%); medical sciences (5.5%); and epidemiology (2.4%). Additionally, 18.9% of respondents indicated that their highest degree was in another (unspecified) field. More than 45% of survey participants held advanced degrees. PN was perceived by 74% of the sample as highly important within the field of nutrition; an additional 19.5% rated the field as important. Most respondents (94%) indicated they are not sufficiently knowledgeable in PN. Only 9.5% received training in nutrigenetics Of these, 73.5% had participated in a one-day seminar and an additional 21.3% were trained in a basic (but not advanced) human genetics course during their academic studies. While 31.4% of the participants replied that they read journal articles in the field of PN annually, most (47.9%) read less frequently than annually. 

Table 2 presents the proportion of correct responses to the knowledge questions on genetics and nutrigenetics. The mean knowledge score was 6.89 ± 1.67, with no difference in the total score detected a cross health professions or workplace settings. 

Only 10% of participants correctly identified the definition of nutrigenetics. Compared to other health professionals, a greater proportion of dietitians in comparison to other professionals correctly recognized the definition of “phenotype” (97.1% vs. 88.1%, respectively); additionally, a greater proportion identified a disease that is not multifactorial (83.3% vs. 62.7%, respectively). 

A multivariate regression model was developed to examine the characteristics of participants in the highest knowledge score tertile (nine points and higher). The model indicated that the only significant predictor was education. Individuals with a MSc degree or higher were 4.4 times more likely to receive a knowledge score of nine and above (Table 3). 

## 4. Discussion

This study investigated the level of knowledge in nutrigenetics and attitudes towards nutrigenetics knowledge and counselling among health care professionals participating in the leading annual nutrition conference in Israel. We found that the overwhelming majority (93.5%) of the participants perceived PN counselling as highly important within the field of nutrition; however, the mean knowledge survey score was 6.89 ± 1.67 out of 12, with substantial variation across different genetic themes. Similar levels of knowledge were reported in previous studies, which showed that dietitians had insufficient knowledge in nutritional genomics and were not confident in their ability to clinically implement it [23]. These findings are also in line with the reports of Whelan et al. [24] and McCarthy et al. [25], showing an average genetic knowledge score of 41% among UK dietitians. The fact that the genetic score findings in our study do not differ significantly from those in other studies performed more than a decade ago, suggest that genetic education to improve dietitians’ knowledge was not implemented. Furthermore, the fact that similar results regarding the level of dietitians’ knowledge in genetics are shown across time and countries indicate that this challenge is universal.

In our study, the single predictor for higher knowledge score was education. No difference in the total score was detected across health professions or workplace settings. 

Israeli registered dietitians scored similarly to their colleagues in the USA, UK, and Australia [10], though variations across questions were significant. For example, while 90.1% of professionals at the Collins et al. sample selected the correct definition of chromosome, only 46.1% of the registered dietitians in our sample replied correctly. Conversely, 92.5% of the registered dietitians in our sample identified the correct definition of allele compared to only 43.2% of the Collins et al. sample. These differences may reflect differences in university curricula across countries.

The need to incorporate advanced courses in nutrigenetics in the already packed health sciences and particularly dietetics curricula has been both identified and challenged [26]. The challenge appears to reflect the multidisciplinary complexity of nutrigenetics, encompassing many fields including genetics, nutrition, statistics, ethics, and law. The challenge of incorporating advanced genetics education has been identified across many health disciplines, including cardiology [27], primary care [28], and occupational therapy [29]. Advanced genetics education was also proved difficult among medical students [30], among others. Dieticians are the primary and best qualified health care professionals for provision of nutrition therapy. As such, they particularly need to obtain further education and training in the field of nutrigenetics. Few studies evaluating the knowledge and perception of nutrigenetics among health care professionals in general and dieticians in particular have been reported. Our study findings indicate that dietitians would like to enhance their education and training in nutrigenetics.

PN should be perceived, understood, and utilized in a larger context, determining the individual’s response to diet, which results from the interaction of metabolic, environmental, social, and genetic factors. Ultimately, with the evolution of high-throughput “omics” technologies, genetic information can be incorporated to predict individual risk factors of rare and common diseases, to incorporate knowledge regarding responders and nonresponders to specific nutritional patterns and to adjust general nutrition based on one’s DNA. 

Another important consideration is consumer attitudes towards nutrigenetics. With dietitians being the first line of contact with the public regarding nutrigenetics inquiries (regardless of whether or not they are legally entitled to advise on these topics), it is necessary for dietitians to at least be familiar with the field of nutrigenetics. In fact, several studies focusing on this issue found positive attitudes towards nutrigenetics and its beneficial use, in parallel with concerns regarding confidentiality issues [31,32,33,34]. The importance of consumer attitudes and perception toward incorporation of nutrigenetics as part of nutritional therapy was demonstrated in the Food4Me study, where participants were genotyped and categorized as risk carriers (AA/AT) or nonrisk carriers (TT) of the fat-mass and obesity-associated (FTO) gene. Regardless of genotype, participants were randomized to one of four intervention groups: Dietary, phenotype, genotype, or control. Only participants in the genotype intervention group were informed of their FTO risk level. Compared to controls, participants with the FTO risk allele had greater reduction in measures of adiposity [35,36]. In contrast, knowledge of the MTHFR genotype (one of the factors associated with cardiovascular disease risk) was not associated with a change in dietary folate intake among Food4Me study participants [18]. 

The effects and magnitude of gene-nutrition interactions is being rapidly revealed. This information will eventually lead to reliable, scientifically sound individualized nutritional recommendations. Among the many remaining challenges are the need for strengthening the science, training professional personnel, as well as improving information delivery and public education. Thus, as the “genetic revolution” accelerates in a rapid pace, health professionals, including registered dieticians, are obligated to acquire sufficient and proper knowledge in genetics in order to fit to the new genetic paradigm. Despite this, the majority of health professionals surveyed in the present study expressed lack of knowledge in nutrigenetics but nevertheless indicated an eagerness to step into this field. 

While at present the direct implications and applications of nutrigenetics in clinical dietetics are limited, commercial direct to consumer nutrigenetics information is already readily available, and clinical dietitians should be sufficiently knowledgeable in this field, even if to point out the extreme limitations of the clinical relevance of the information available at present. Moreover, the pace of acquired knowledge in the field is high and in coming years is expected to be of routine relevance to clinical dietetics, and thus knowledge and education in this field is of importance in preparing clinical dietitians for this new era. 

Findings herein must be understood in the context of study limitations. First, the data are cross-sectional and, as such, causality cannot be inferred. Associations between sociodemographic or professional characteristics and level of knowledge are correlational only. Second, although this study tested knowledge objectively, it was limited to 12 questions and focused only on the theory of genetics and nutritional genomics and, as such, was not measured exhaustively. Finally, the study sample was drawn from registered attendees of a large nutrition conference. There is no way to determine the extent to which survey respondents are similar to or differ from the population of dietitians and other health care workers. This of course limits external validity. Nevertheless, the study population is large, which improves response prevalence estimates.

## 5. Conclusions 

The field of nutrigenetics is developing rapidly in parallel to the huge advances in human genetics discoveries over the last decade, enabling major improvements in prevention, diagnosis, and treatment of many rare and common diseases. The enormous progress in biotechnology and bioinformatics enables economically-affordable sequencing of one’s DNA and thus enables fast incorporation of genetic data into the clinical world. However, nutrigenetics is a complex field involving multiple factors and interactions, many of which are not yet well known or established. Thus, incorporation and implementation of nutrigenetics into practical dietetics should be critically examined and properly translated by nutrigenetics experts to the public. Similar to other health professions, the need for proper education in the field of human genetics was identified in the present study. Our findings clearly indicate that health professionals, and particularly dietitians, recognize the importance of acquiring knowledge in nutrigenetics. Only by acquiring the necessary scientific framework can dietitians accurately translate this knowledge into clinical practice and maintain their status as the nutrition expert. 

## Figures and Tables

**Table 1:** Characteristics of survey participants (*n* = 169).

Characteristic	Result
Age (years)	37.9 ± 10.4
Sex *n,* (% female)	159 (94.1)
Profession *n*, (%)	
Dietitian	102 (60.4)
Student/intern	21 (12.4)
Food technologist	20 (11.8)
Nurse	4 (2.4)
Physician	2 (1.2)
Other	20 (11.8)
Highest level of education *n*,(%)	
BSc, BA	77 (45.6)
MSc, MA, MPH, MBA	74 (43.8)
PhD	9 (5.3)
MD	1 (0.6)
Undergraduate student	8 (4.7)
Employment setting *n*, (%)	
Private clinic	14 (8.3)
Hospital	42 (24.9)
Health maintenance organization clinic	19 (11.2)
Academia	5 (3.0)
Private company conducting clinical research	11 (2.4)
Food industry	21 (12.4)
Drug industry	2 (1.2)
Unemployed	7 (4.1)
Other	47 (27.8)
Work experience, years (mean ± SD)	10.7 ± 10.5

**Table 2:** Professionals’ attitudes, training, and knowledge of genetics and nutrigenetics (*n* (%)).

Multiple Choice Question	Overall *n* = 169 *n*, (%)	Dietitians *n* = 102 *n*, (%)	Other Professions * *n* = 67 *n*, (%)	*p*-Value
A ‘gene’ is?	136 (80.5)	81 (79.4)	55 (82.1)	0.4
A ‘chromosome’ is?	84 (49.7)	47 (46.1)	37 (55.2)	0.2
An ‘allele’ is?	160 (94.7)	98 (96.1)	62 (92.5)	0.2
‘Genotype’ is?	47 (27.8)	32 (31.4)	15 (22.4)	0.1
‘Phenotype’ is?	158 (93.5)	99 (97.1)	59 (88.1)	0.02 **
A ‘polymorphism’ is?	99 (58.6)	61 (59.8)	38 (56.7)	0.4
A ‘mutation’ is?	91 (53.8)	53 (53.9)	36 (53.7)	0.5
What condition is not associated with change in MTHFR C677T?	56 (33.1)	35 (34.3)	21 (31.3)	0.4
Which of the following diseases is not multifactorial?	127 (75.1)	85 (83.3)	42 (62.7)	0.02 **
‘Nutrigenetics’ is?	17 (10.1)	13 (12.7)	4 (6.0)	0.2
In case of Lactose intolerance, which of the following genetic conditions can occur?	95 (56.2)	61 (59.8)	34 (50.7)	0.2
Can a child with genetic illness be the decedent of healthy parents?	155 (91.7)	92 (90.2)	63 (94.0)	0.4
Total knowledge score (mean ± SD)	6.89 ± 1.67	7.08 ± 1.55	6.63 ± 1.81	0.08
**Attitudes and training**				
How important is personalized nutrition within the field of nutrition?				
Very important	126 (74.5)	75 (76.1)	51(73.5)	0.58
Important	33 (19.5)	19 (18.6)	14 (20.9)	
Less important	9 (5.3)	7 (6.9)	2 (3.0)	
Not important	1 (0.6)	1 (1.0)	0 (0.0)	
Are you sufficiently knowledgeable in personalized nutrition?				
No *n*, (%)	10 (5.9)	9 (5.3)	(0.6)	0.05
Have you obtained any training in nutrigenetics?				
Yes *n*, (%)	16 (9.5)	13 (7.7)	3 (1.8)	0.07
How frequently are you updated in professional literature regarding nutrigenetics?				
Weekly	9 (5.3)	5 (4.9)	4 (6.0)	0.46
Monthly	26 (15.4)	13 (12.7)	13 (19.4)	
Annually	53 (31.4)	36 (35.3)	17 (25.4)	
Less than annually	81 (47.9)	48 (47.1)	33 (49.3)	

* Other health professions comprised 39.6% of the sample, of them: Students and interns (12.5%), food technologist (11.9%), nurses (1.9%), physicians (1.2%), and others (11.5%).** *p* < 0.05.

**Table 3:** Factors associated with total knowledge score higher than nine points in multivariable logistic regression analysis.

	Odds Ratios	95% CI	*p* Value
Age (years)	1.01	0.97–1.06	0.51
Sex (women vs. men)	1.64	0.18–14.88	0.66
Profession (dietitians vs. other)	0.65	0.25–1.71	0.38
Academic Degree (MSc and above vs. BSc and below)	4.03	1.34–12.11	0.013
Employment setting (clinical vs. other)	1.29	0.48–3.46	0.61
Constant	0.03		0.14

## Supplementary Materials

The following are available online at https://www.mdpi.com/2072-6643/12/2/335/s1. File S1: Knowledge attitudes and training survey of nutrigenetics.
