# Supplementary material for: Knowledge and Attitudes Towards Nutrigenetics: Findings from the 2019 Unified Forces Preventive Nutrition Conference (UFPN)
Source: Nutrients. 2020 Jan 27;12(2):335. doi: 10.3390/nu12020335 (PMC7071140; doi:10.3390/nu12020335)
Supplement: Supplementary file 1 [file nutrients-12-00335-s001.pdf]

Supplement 1: knowledge attitudes and training survey of nutrigenetics

Instructions: in each of the following questions, please choose the most accurate reply

1. A ‘gene’ is:
    a. A segment of DNA containing information on specific hereditary trait
    b. An RNA molecule
    c. A segment of protein containing information on specific hereditary trait
    d. A DNA segment
2. A ‘chromosome’ is:
    a. Genetic information organized in a structure of the same size in all humans
    b. A large protein which contains sequences that binds to the DNA
    c. A combination of DNA, RNA and proteins that encompasses all the genetic information of humans
    d. Genetic information, that is grouped in a particular site of the genome
3. An ‘allele’ is:
    a. A distinct copy of a gene
    b. A maternal copy of a gene
    c. A paternal copy of a gene
    d. One of two copies of a gene
4. ‘Genotype’ is:
    a. All genes in the genome
    b. All sequences of DNA in the cell
    c. All proteins of the cell
    d. All genes expressed in the fetus
5. ‘Phenotype’ is:
    a. The external expression of a hereditary trait
    b. The structure of a gene
    c. The nucleotide sequence within a gene
    d. None of the possibilities above
6. A ‘polymorphism is:
    a. A difference in the number of genes between humans
    b. A difference in the genome sequence across humans
    c. A difference in the genome sequence between cells of the same individual
    d. A difference in the number of active proteins across humans
7. A ‘mutation’ is:
    a. A change in the DNA sequence within a specific gene
    b. A change in the DNA sequence which leads to a disease
    c. A change in the DNA sequence that does not necessarily lead to a change in protein
    d. Deletion of a gene within an individual’s genome

8. What condition is not associated with change in MTHFR C677T?

   a. Neural tube defect
   b. Elevated homocysteine levels
   c. Elevated methionine levels
   d. Elevated lipids levels

9. Which of the following diseases is not a multifactorial disease?
   a. Cystic fibrosis
   b. Ischemic heart disease
   c. Type 2 diabetes mellitus
   d. Dyslipidemia

10. 'Nutrigenetics' is?
    a. Nutrition adjusted to the human genome
    b. Nutrition adjusted to the human genome, environment and the interaction between them
    c. Nutrition adjusted to the family's background
    d. Nutrition adjusted to diseases
    e. All of the above

11. Regarding the genetic variation causing Lactose intolerance, which of the following genetic conditions can occur?
    a. Homozygote to the change
    b. Heterozygote to the change
    c. No change
    d. All of the above

12. Can a child with genetic illness be the decedent of healthy parents?
    a. Yes
    b. No
    c. Yes, only in a specific environment
    d. Only if the parents are relatives

13. In your opinion, how important is personalized nutrition within the field of nutrition
    a. Very important
    b. Important
    c. Less important
    d. Not important
14. Are you sufficiently knowledgeable in personalized nutrition?
    a. Yes
    b. No
15. Have you obtained any training in nutrigenetics?
    a. Yes
    b. No

16. If you replied yes in question 15, which training have you obtained in nutrigenetics?
    a. A short workshop (up to one day)
    b. A course (30 academic hours and above)
    c. A degree
17. Do you read professional literature in the field of nutrigenetics?
    a. Yes
    b. no
18. How frequently are you updated in professional literature regarding nutrigenetics?
    a. Weekly
    b. Monthly
    c. Annually
    d. Less than annually
